# Office-Based Carpal Tunnel Release With Ultrasound Guidance: 6-month Outcomes From the Multicenter ROBUST Trial

**DOI:** 10.1016/j.jhsg.2023.12.005

**Published:** 2024-02-19

**Authors:** Ashley L. Pistorio, Victor M. Marwin, Paul D. Paterson, Randall D. Alexander, Johnny T. Nelson, Larry E. Miller

**Affiliations:** ∗ Department of Plastic Surgery, Kirk Kerkorian School of Medicine, University of Nevada Las Vegas, Las Vegas, NV; † Bluegrass Orthopaedics, Lexington, KY; ‡ Vero Orthopaedics, Vero Beach, FL; § Georgia Hand, Shoulder & Elbow, Atlanta, GA; ‖ The Bone and Joint Surgery Clinic, Raleigh, NC; ¶ Miller Scientific, Johnson City, TN

**Keywords:** Carpal tunnel release, CTR-US, Office, ROBUST, Ultrasound

## Abstract

**Purpose:**

The purpose of this study was to determine the safety and effectiveness of office-based carpal tunnel release with ultrasound guidance (CTR-US).

**Methods:**

In this prospective multicenter observational study, patients were treated with CTR-US in an office setting. Outcomes were time to resume normal daily activities, time to return to work, Boston Carpal Tunnel Questionnaire Symptom Severity Scale and Functional Status Scale scores, Michigan Hand Questionnaire, Numeric Pain Scale, EuroQoL-5 Dimension 5-Level score, procedure satisfaction, and adverse events over 6 months.

**Results:**

A total of 149 participants (226 hands) from seven centers underwent office-based CTR-US. The mean age was 58 years, 52% were women, and 68% were employed. The mean incision length was 5 mm, 52% had simultaneous bilateral procedures, and wide-awake local anesthesia no tourniquet was used in all cases. All procedures were completed as planned, with no conversions to open repair and mean intraoperative pain severity of 1.6 ± 1.5. The median time to resume normal activities was 2 days (interquartile range: 1–4 days) and return to work was 4 days (interquartile range: 1–5 days). Over 6 months, Boston Carpal Tunnel Questionnaire Symptom Severity Scale decreased by a mean of 1.7 points, Boston Carpal Tunnel Questionnaire Functional Status Scale decreased by 1.1 points, Michigan Hand Questionnaire Global score increased by 35 points, Numeric Pain Scale decreased by 3.7 points, and EuroQoL-5 Dimension 5-Level score increased by 0.11 points. At 6 months, 94% reported procedure satisfaction. Unilateral and simultaneous bilateral procedures were similarly effective. There was one (0.4%) adverse event, a nerve contusion treated with neurolysis and nerve wrap where the patient fully regained normal function within 7 weeks. There were no revisions for persistent or recurrent carpal tunnel syndrome symptoms.

**Conclusions:**

Office-based CTR-US, performed either unilaterally or as simultaneous bilateral procedures, is well tolerated with a low complication rate and associated with rapid recovery, sustained improvement in symptoms and function, and high procedure satisfaction.

**Type of study/level of evidence:**

Therapeutic III.

Carpal tunnel syndrome (CTS) is a common compression neuropathy of the median nerve at the wrist, affecting approximately 2%–4% of the general population.^1^^,^^2^ An estimated 600,000 carpal tunnel release (CTR) procedures are performed in the United States annually,^3^ with 3% of adults projected to undergo CTR during their lifetime.^4^ Traditionally, CTR procedures have been performed in operating rooms or ambulatory surgery centers (ASCs). However, since facility costs are the primary expenditure associated with CTR,^5^^,^^6^ interest has developed in office-based CTR.^7^^,^^8^ Potential benefits of office-based CTR include lower facility costs, increased practice efficiency, patient convenience, faster patient recovery, and higher patient satisfaction compared with CTR in hospital/ASC facilities.^9^, ^10^, ^11^, ^12^ Still, office-based CTR remains underused.^7^^,^^8^^,^^12^ Concerns about transferring CTR from dedicated facilities to office settings include the potential for higher complication rates and inferior outcomes. However, accumulating evidence suggests that these concerns may be unfounded.^9^, ^10^, ^11^, ^12^ Further study on the effects of CTR procedure types and operative settings on patient outcomes is warranted.

Carpal tunnel release with ultrasound guidance (CTR-US) is well suited for the office setting because it uses a small (< 1 cm) wrist incision to access and divide the transverse carpal ligament (TCL) under continuous ultrasound (US) visualization. This provides an expanded field of view compared with endoscopic and mini-open techniques. Although numerous studies have demonstrated the safety and effectiveness of CTR-US,^13^, ^14^, ^15^, ^16^, ^17^, ^18^, ^19^ less is known about the outcomes of office-based CTR-US. Office-based CTR-US was first described in a single-center study by Chappell et al.^13^ Among 37 hands followed for 6 to 10 weeks after the procedure, statistically significant reductions were noted in median nerve cross-sectional area, with improvements in symptom severity and function and no reported complications. Bergum et al^14^ corroborated these results in their prospective, single-center series of 123 hands where office-based CTR-US provided statistically significant and clinically important improvements in symptoms and function over 1 year with no intraoperative complications or reoperations. Yet, it remains unclear whether these single-center office-based CTR-US results can be reproduced across multiple practices. Therefore, this prospective multicenter observational study aimed to determine the safety and effectiveness of office-based CTR-US.

## Materials and Methods

### Study design

This prospective multicenter observational study enrolled subjects undergoing unilateral or simultaneous bilateral CTR-US procedures in an office setting. The trial was registered on ClinicalTrials.gov (NCT05675046) before subject enrollment. The study protocol was approved by a central institutional review board (Western-Copernicus Group Institutional Review Board). Subjects provided written informed consent before participation. An independent data safety monitoring board provided study oversight. Participating centers and oversight committee members are listed in Supplementary Table 1 (available online on the Journal’s website at https://www.jhsgo.org). The study followed the Strengthening the Reporting of Observational Studies in Epidemiology reporting guidelines.^20^

### Participants and eligibility criteria

Patients with unilateral or bilateral idiopathic CTS considered potential candidates for CTR were evaluated for the study. Patients underwent a preoperative clinical examination and diagnostic US of the median nerve. The inclusion criteria were similar to those used in previous CTR-US studies performed in various settings.^16^^,^^21^ Key eligibility criteria were a clinical diagnosis of unilateral or bilateral idiopathic CTS, CTS-6 score of at least 12 in the target hand(s),^22^ median nerve cross-sectional area of at least 10 mm^2^ at the carpal tunnel inlet region of the target hand(s),^22^^,^^23^ and persistent symptoms despite nonsurgical treatment. Key exclusion criteria were systemic inflammatory disease, previous CTR in the target hand, previous infection in the target hand, recent (<6 weeks) corticosteroid injection in the target wrist or hand, or planned surgical or interventional procedure on the contralateral wrist or hand within 3 months. For patients with bilateral CTS, eligibility criteria were evaluated separately for each hand where applicable. Complete eligibility criteria are listed in Supplementary Table 2 (available online on the Journal’s website at https://www.jhsgo.org). Eligible subjects were enrolled and scheduled for CTR-US within 30 days.

### Procedure

All procedures were performed using field sterility in office settings.^24^^,^^25^ Per the Centers for Medicare and Medicaid Services policy, an office setting was defined as locations where health professionals routinely provide health examinations, diagnoses, and treatments of illness or injury on an ambulatory basis, excluding hospitals, ambulatory surgical centers, skilled nursing facilities, military treatment facilities, community health centers, state or local public health clinics, or intermediate care facilities.^26^ All subjects underwent CTR-US using the UltraGuideCTR device (Sonex Health, Inc). The entire procedure was performed under continuous real-time US guidance. A small (<1 cm) incision was made near the proximal wrist crease to insert the hand-held device into the carpal tunnel. The blunt device tip is designed for enhanced US visibility and safety with two inflatable balloons bordering a central retractable retrograde cutting blade. Once inserted, the tip was positioned deep and distal to the TCL. The balloons were inflated with sterile saline to create and maintain space within the carpal tunnel. Ultrasound confirmed the safe positioning of the tip relative to the surrounding anatomy. The cutting blade was activated, and the TCL was transected in a retrograde direction. After transection, the blade was retracted, the balloons were deflated, and the device was removed. Finally, the TCL was probed using US guidance to ensure complete release. The incisions were typically closed without sutures. Complete procedural steps for performing CTR-US with this device have been previously described.^14^^,^^16^

### Outcomes

Postoperative data were collected remotely using a validated electronic data capture system (Viedoc). Subjects completed follow-up assessments daily for the first 14 days after the procedure and then at 1, 3, and 6 months. Subjects remain in follow-up for 2 years (Supplementary Table 3, available online on the Journal’s website at https://www.jhsgo.org). Study outcomes were time to resume normal daily activities, time to return to work, Boston Carpal Tunnel Questionnaire Symptom Severity Scale (BCTQ-SSS) and Boston Carpal Tunnel Questionnaire Functional Status Scale (BCTQ-FSS) scores, Michigan Hand Questionnaire (MHQ) scores, Numeric Pain Scale scores, EuroQoL-5 Dimension 5-Level (EQ-5D-5L) scores, procedure satisfaction, and device- or procedure-related adverse events through 6 months. The primary end point was the change in BCTQ-SSS at 3 months where we hypothesized that the improvement would be statistically significant compared with baseline.

Patients self-reported time to resume normal daily activities outside of work. Employed patients self-reported time to return to work in any capacity. The BCTQ-SSS and BCTQ-FSS scores range from 1 to 5, with higher scores indicating worse CTS-related symptom severity and functional status.^27^ The MHQ is a 37-question hand-specific questionnaire that assesses overall hand function, activities of daily living, work performance, pain, aesthetics, and satisfaction.^28^ The MHQ provides a Global score and Domain scores (scored 0–100), where higher values represent better outcomes. Subjects rated hand/wrist pain severity on a 0–10 Numeric Pain Scale. Health-related quality of life was assessed using the EQ-5D-5L questionnaire (scored 0–1).^29^ The minimal clinically important difference after CTR-US was defined as a change in −1.14 points for BCTQ-SSS,^30^ −0.74 points for BCTQ-FSS,^30^ −2 points for Numeric Pain Scale,^31^ 9 points for the MHQ Global score,^32^ and 0.09 points for the EQ-5D-5L.^33^ Procedure satisfaction was self-reported on a 5-point Likert scale ranging from very dissatisfied to very satisfied. Satisfaction was defined as participants reporting being satisfied or very satisfied.

Study investigators classified adverse events for seriousness and relationship to the device or procedure. An independent medical reviewer evaluated and adjudicated all adverse events based on classification, seriousness, and relationship. Discrepancies between the investigator and medical reviewer were resolved through discussion, with the judgment of the medical reviewer considered final.

### Statistical analysis

Baseline continuous data were reported as means and SDs or medians and interquartile ranges (IQRs). Counts and percentages were used for categorical data. The time to resume normal daily activities and return to work among employed individuals were reported using medians and IQRs and plotted using a cumulative incidence function. Continuous outcomes measured longitudinally were analyzed with linear mixed-effects models. Longitudinal changes in patient-reported outcomes were also reported with the standardized effect size statistic where absolute values of 0.2, 0.5, 0.8, and 1.0 are considered small, medium, large, and very large effect sizes, respectively. Baseline comparisons of unilateral and simultaneous bilateral cases were made using Wilcoxon rank-sum tests for continuous variables and Fisher exact tests for categorical variables. For longitudinal outcomes comparing unilateral and simultaneous bilateral cases, the linear mixed-effects models included an interaction term between time and a binary indicator for the number of treated hands. Adverse events were reported using counts, percentages, and exact 95% confidence interval (CI). All significance testing was two-sided, and the results were considered statistically significant if the *P* value was less than .05.

## Results

Between February and June 2023, office-based CTR-US was performed on 149 participants (226 hands) at seven centers in the United States. Most (six of seven) centers were private practices, and all investigators were fellowship-trained hand surgeons. Among the seven surgeons, six performed fewer than 100 CTR-US procedures before the trial (median: 11 cases). Four surgeons had performed multiple office-based CTR-US procedures, one had performed a single office-based CTR-US, and three had no prior experience with office-based CTR-US.

The mean patient age was 58 years, 52% were women, and 68% were employed. The most common comorbidities were obesity (62%), hypertension (35%), anxiety (22%), thyroid disease (17%), and depression (17%). Among eight (5%) patients using anticoagulants before surgery, only one was instructed to modify the medication regimen before the procedure. Among employed patients, 59% held desk-based positions, 23% performed light manual labor, and 19% were heavy manual laborers (Table 1). Most (60%) patients experienced CTS symptoms for more than 2 years, the mean CTS-6 score was 19.1 ± 3.9, the median nerve cross-sectional area was 15.9 ± 4.5 mm^2^, and 64% had bilateral disease at the time of their procedure (Table 2). Baseline values for key patient-reported outcomes were 3.0 ± 0.6 points for BCTQ-SSS, 2.3 ± 0.8 points for BCTQ-FSS, 55 ± 18 points for MHQ Global score, 4.4 ± 2.5 points for the Numeric Pain Scale, and 0.78 ± 0.11 points for the EQ-5D-5L.

**Table 1:** Demographic Characteristics of Patients Treated With Office-Based Carpal Tunnel Release With US Guidance

Variable	Value∗
**Demographics**	
Age (y)	58 ± 13
Women	51.7% (77/149)
Race	
White	90.4% (132/146)
Black/African American	4.1% (6/146)
Asian	1.4% (2/146)
Other	4.1% (6/146)
Body mass index (kg/m^2^)	32 ± 7
**Medical history**	
Obesity	62.4% (93/149)
Hypertension	34.9% (52/149)
Anxiety	22.1% (33/149)
Thyroid disease	17.4% (26/149)
Depression	16.8% (25/149)
Current tobacco use†	10.1% (15/149)
Hyperlipidemia	9.4% (14/149)
Diabetes	8.1% (12/149)
Anticoagulant use	5.4% (8/149)
Opioid use	4.7% (7/149)
Fibromyalgia	4.0% (6/149)
Polyneuropathy/other nerve disorder	2.0% (3/149)
**Employment history**	
Employed	68.5% (102/149)
Work activity	
Desk	58.8% (60/102)
Light manual activity	22.5% (23/102)
Heavy manual activity	18.6% (19/102)

∗ Values are mean ± SD or percent (n/N).

† Includes any smoking or nicotine products.

**Table 2:** Clinical Characteristics of Patients Treated With Office-Based Carpal Tunnel Release With US Guidance

Variable	Value∗
**Carpal tunnel syndrome history**	
Symptom duration	
3–6 m	9.5% (14/148)
>6 mo–1 y	14.2% (21/148)
>1–2 y	16.2% (24/148)
>2 y	60.1% (89/148)
Bilateral carpal tunnel syndrome	63.8% (95/149)
CTS-6 total score	19.1 ± 3.9
Median nerve cross-sectional area (mm^2^)	15.9 ± 4.5
**Patient-reported outcomes**	
BCTQ-SSS (1–5 scale)	3.0 ± 0.6
BCTQ-FSS (1–5 scale)	2.3 ± 0.8
MHQ Global score (0–100 scale)	55 ± 18
Numeric Pain Scale (0–10 scale)	4.4 ± 2.5
EQ-5D-5L (0–1 scale)	0.78 ± 0.11

∗ Values are mean ± SD or percent (n/N).

Unilateral procedures were performed in 48% of the patients and simultaneous bilateral procedures in 52% (comprising 77 of the 95 patients with bilateral disease; Table 3). All procedures were completed as planned with no conversions to open repair. The wide-awake local anesthesia no tourniquet (WALANT) technique was used in all cases, with most (79%) using epinephrine. The mean surgical incision length in the wrist was 5 mm (range: 3–8 mm). The procedures were well tolerated overall; the mean intraoperative pain severity was 1.6 ± 1.5, and 79% of patients reported that the anesthetic injection was the most painful part of the procedure.

**Table 3:** Procedural Details in Patients Treated With Office-Based Carpal Tunnel Release With US Guidance

Variable	Value∗
Anesthesia	
Local with epinephrine	79.2% (118/149)
Local without epinephrine	20.8% (31/149)
Regional	0%
Monitored anesthesia care	0%
General	0%
Incision length (mm)	5 ± 1
Dominant hand treated	82.6% (123/149)
Simultaneous bilateral procedure	51.7% (77/149)
Suture-free wound closure	98.7% (147/149)

∗ Values are mean ± SD or percent (n/N).

The median time to resume normal daily activities was 2 days (IQR: 1–4; Fig. 1). The percentage of patients resuming normal daily activities was 35% within 1 day, 58% within 2 days, 70% within 3 days, 85% within 5 days, and 98% within 14 days. Among employed patients, the median time to return to work was 4 days (IQR: 1–5; Fig. 2). The percentage of patients returning to work was 25% within 1 day, 40% within 2 days, 49% within 3 days, 77% within 5 days, and 94% within 14 days. Comparing patients with desk-based, light-manual, and heavy-manual jobs, the median time to return to work was 3, 4, and 4 days, respectively.

**Figure 1:**
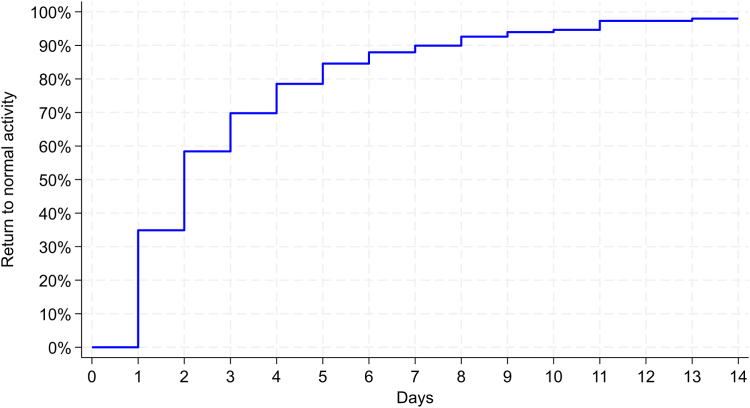
Time to resume normal daily activities following office-based carpal tunnel release with US guidance. The median time to resume normal daily activities was 2 days (IQR: 1–4 days). The percentage of patients resuming normal daily activities was 35% within 1 day, 58% within 2 days, 70% within 3 days, 85% within 5 days, and 98% within 14 days.

**Figure 2:**
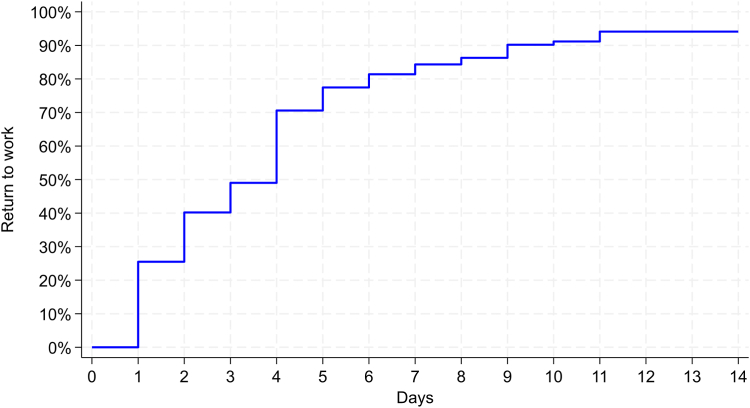
Time to return to work following office-based carpal tunnel release with US guidance. The median time to return to work was 4 days (IQR: 1–5 days). The percentage of patients returning to work was 25% within 1 day, 40% within 2 days, 49% within 3 days, 77% within 5 days, and 94% within 14 days.

**Figure 3:**
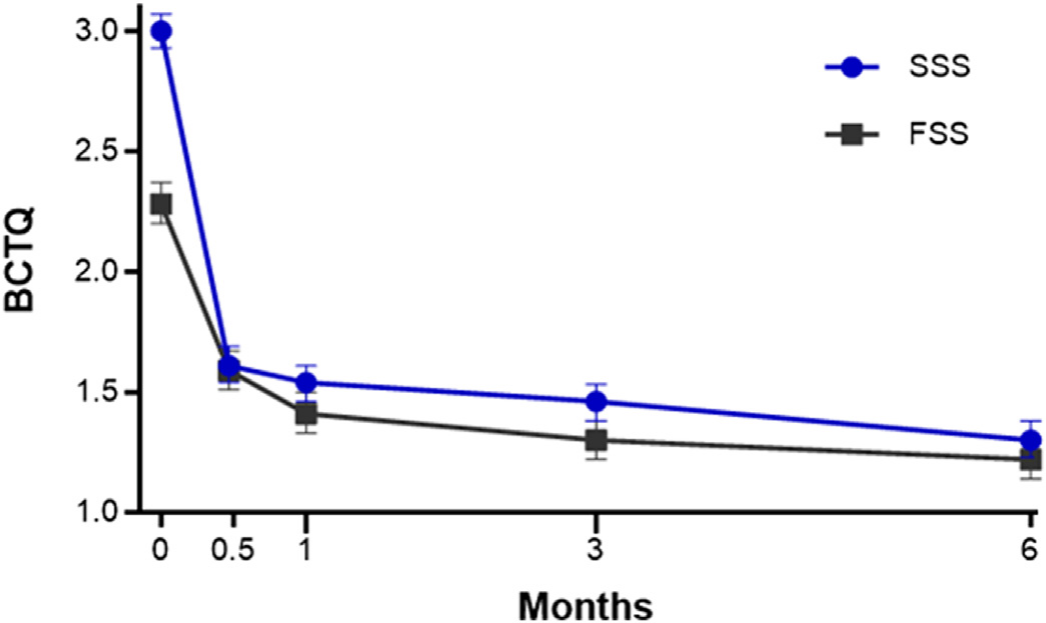
BCTQ-SSS and BCTQ-FSS scores over 6 months following office-based carpal tunnel release with US guidance. At 6 months, the mean change from baseline was − 1.7 points for BCTQ-SSS and −1.1 points for BCTQ-FSS. At all follow-up intervals, the mean change was statistically significant compared with baseline (all *P* < .001) and exceeded the minimal clinically important difference of a 1.14-point decrease for BCTQ-SSS and a 0.74-point decrease for BCTQ-FSS, respectively. Error bars represent 95% CI.

**Figure 4:**
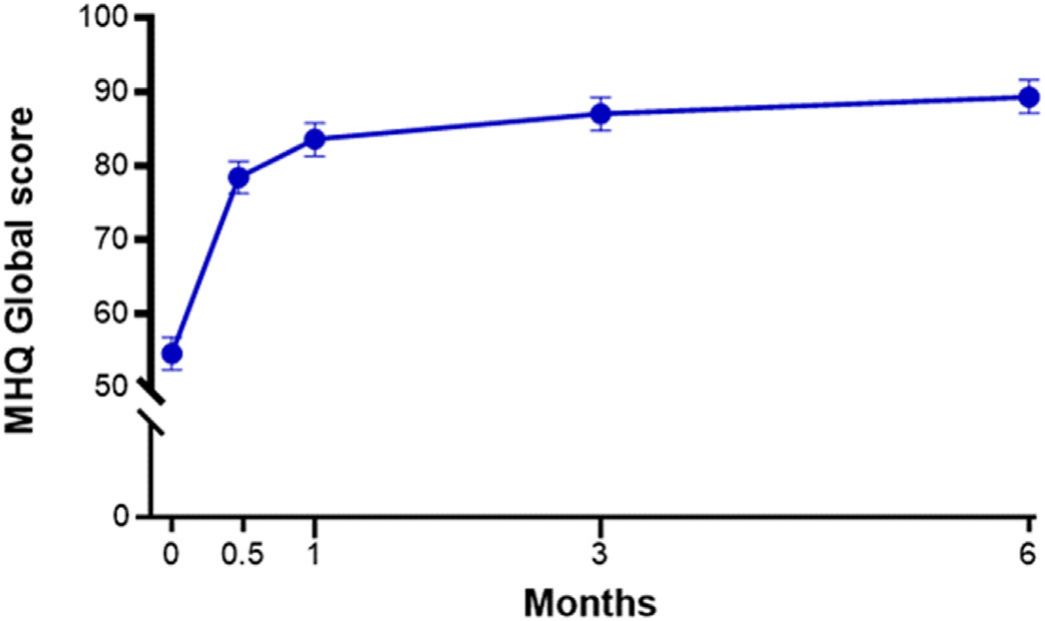
MHQ Global scores over 6 months following office-based carpal tunnel release with US guidance. At 6 months, the mean change from baseline was 35 points. At all follow-up intervals, the mean change was statistically significant compared to baseline (all *P* < .001) and exceeded the minimal clinically important difference of a 9-point increase. Error bars represent 95% CI.

**Figure 5:**
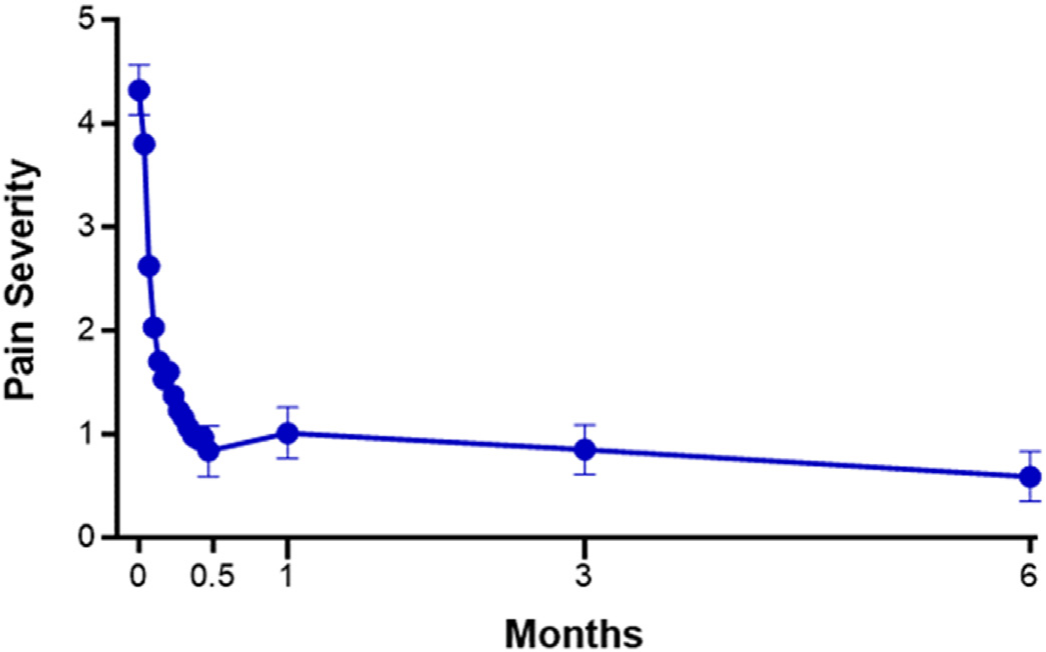
Numeric Pain Scale over 6 months following office-based carpal tunnel release with US guidance. At 6 months, the mean change from baseline was −3.7 points. At all follow-up intervals, the mean change was statistically significant compared with baseline (all *P* < .001) and, from postprocedure day 3 and thereafter, exceeded the minimal clinically important difference of a 2-point decrease. Error bars represent 95% CI.

**Figure 6:**
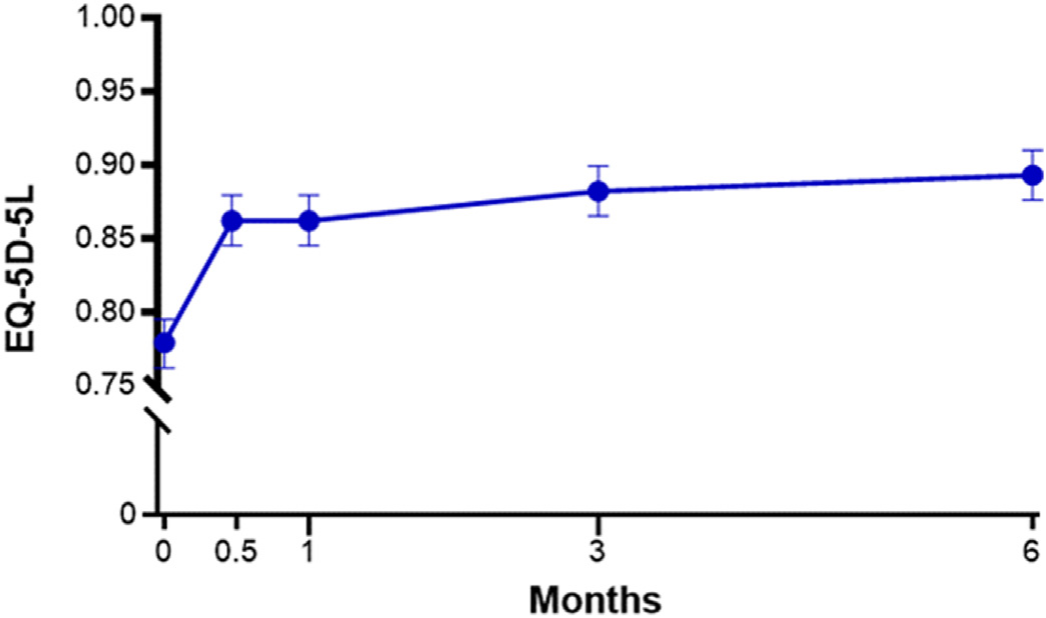
EQ-5D-5L scores over 6 months following office-based carpal tunnel release with US guidance. At 6 months, the mean change from baseline was 0.11 points. At all follow-up intervals, the mean change was statistically significant compared with baseline (all *P* < .001) and exceeded the minimal clinically important difference of a 0.09-point increase. Error bars represent 95% CI.

Outcome data were provided by 97% of patients at the 6-month follow-up. The primary end point of the study was met as the 3-month change in BCTQ-SSS was −1.5 (95% CI: −1.6 to −1.4; *P* < .001). Over 6 months, BCTQ-SSS and BCTQ-FSS (Fig. 3), MHQ Global score (Fig. 4), Numeric Pain Scale (Fig. 5), and EQ-5D-5L (Fig. 6) significantly improved, with all mean changes exceeding the minimal clinically important difference. The mean change over 6 months was −1.7 points for BCTQ-SSS, −1.1 points for BCTQ-FSS, 35 points for MHQ Global score, −3.7 points for the Numeric Pain Scale, and 0.11 points for the EQ-5D-5L (all *P* < .001). Additionally, all MHQ domain scores significantly improved over 6 months (all *P* < .001; Table 4). The standardized effect size was 3.2 for BCTQ-SSS, 1.9 for BCTQ-FSS, 2.3 for MHQ Global score, 1.8 for the Numeric Pain Scale, and 1.04 for the EQ-5D-5L, all of which were above the threshold of 1.0 for defining a very large treatment effect. At 6 months, 94% of patients reported satisfaction with the CTR-US procedure. Comparing patients treated with unilateral or simultaneous bilateral CTR-US, baseline characteristics were comparable, and the procedures were similarly effective with only minor differences between the subgroups (Table 5).

**Table 4:** Michigan Hand Questionnaire Global and Domain Scores in Patients Treated With Office-Based Carpal Tunnel Release With US Guidance∗

Variable	Baseline	2 wk	1 mo	3 mo	6 mo
Global	55	78	84	87	89
Overall	55	80	81	86	89
Activities of daily living	58	74	79	82	83
Work	58	68	82	89	91
Aesthetics	77	92	92	93	93
Satisfaction	35	85	84	86	90
Pain	44	72	83	85	90

∗ Changes from baseline were statistically significant (*P* < .001) for all domains at all follow-up intervals, and the Global score exceeded the minimal clinically important difference of 9 points. The 95% CI for all mean values are within ±3 points.

**Table 5:** Comparison of Key Patient Characteristics and Outcomes With Unilateral Versus Simultaneous Bilateral Office-Based Carpal Tunnel Release With US Guidance

Variable∗	Unilateral (n = 72)	Simultaneous Bilateral (n = 77)	*P* Value
**Demographics**			
Age (y)	60 ± 13	57 ± 13	.20
Female sex	50.0% (36/72)	53.2% (41/77)	.74
**Medical history**			
Obesity	62.5% (45/72)	62.3% (48/77)	> .99
Hypertension	38.9% (28/72)	31.2% (24/77)	.39
Anxiety	20.8% (15/72)	23.4% (18/77)	.84
Thyroid disease	18.1% (13/72)	16.9% (13/77)	> .99
Depression	19.4% (14/72)	14.3% (11/77)	.51
Current tobacco use	9.7% (7/72)	10.4% (8/77)	> .99
**Employment history**			
Employed	62.5% (45/72)	74.0% (57/77)	.16
Work activity			.24
Desk	60.0% (27/45)	57.9% (33/57)	
Light manual activity	15.6% (7/45)	28.1% (16/57)	
Heavy manual activity	24.4% (11/45)	14.0% (8/57)	
**Carpal tunnel syndrome history**			
CTS-6 total score	19.4 ± 4.2	18.8 ± 3.5	.40
Median nerve cross-sectional area (mm^2^)	15.5 ± 4.5	16.2 ± 4.4	.16
**Outcomes**			
Days to resume normal activity†	2 (1–4)	2 (1–5)	.04
Days to return to work†	3 (1–4)	4 (2–5)	.53
BCTQ-SSS change through 6 mo‡	−1.7 (−1.9, −1.5)	−1.8 (−2.0, −1.6)	.14
BCTQ-FSS change through 6 mo‡	−1.0 (−1.2, −0.8)	−1.0 (−1.2, −0.8)	.27
MHQ Global score change through 6 mo‡	34 (29, 38)	38 (32, 44)	.03
Numeric Pain Scale through 6 mo‡	−4.0 (−4.6, −3.3)	−4.3 (−5.1, −3.5)	.16
EQ-5D-5L change through 6 mo‡	0.10 (0.06, 0.13)	0.14 (0.09, 0.19)	.10

∗ Values are mean ± SD or percent (n/N) unless otherwise specified.

† Values are median (IQR).

‡ Values are mean change (95% CI).

Among the 226 treated hands, 1 (0.4%) adverse event was reported. The patient underwent simultaneous bilateral CTR-US procedures, which were completed uneventfully. On postprocedure day 2, the patient reported new-onset tingling and hypersensitivity in the right ring finger. Surgical re-exploration on postprocedure day 11 revealed a nerve contusion with a small epineurial injury to the ulnar aspect of the median nerve without evidence of fascicular damage. The injury was determined to have been caused by the Freer elevator tip during insertion. Following neurolysis and a nerve wrap, symptoms improved over 7 weeks, with complete recovery of median nerve function and minimal residual tingling in the affected finger. No other adverse events were reported, including anesthesia reactions, infections, bleeding complications, cysts/seromas, or tendon, muscle, or vascular complications. No revisions for persistent or recurrent CTS symptoms were reported.

## Discussion

This study demonstrated that office-based CTR-US is well tolerated with a low complication rate and is associated with rapid recovery, sustained improvement in symptoms and function, and high procedure satisfaction. Despite nearly one in four patients having anxiety, CTR-US using WALANT was effectively used in these patients, highlighting its usefulness even in those who may traditionally prefer general anesthesia. Unilateral and simultaneous bilateral CTR-US were similarly effective, with both subgroups achieving clinically important improvements in all patient-reported outcomes. No serious intraoperative complications such as bleeding, infection, or anesthesia reactions occurred. The results with office-based CTR-US in the current multicenter trial were consistent with those from single-center office-based CTR-US studies,^13^^,^^14^ as well as from previous studies with CTR-US performed across multiple sites of service.^15^, ^16^, ^17^, ^18^, ^19^ Overall, the results of this prospective multicenter trial support the safety and effectiveness of office-based CTR-US.

The rapid recovery observed in this trial, with a median resumption of normal activities in 2 days and return to work in 4 days, is a significant advantage of office-based CTR-US. These milestones are comparable to the results seen with CTR-US across various settings,^16^^,^^21^ suggesting that CTR-US expedites return to activity and work and that these results may be independent of the operative setting. Further, these results are faster than the meta-analysis-derived return to work estimates of 18 days for endoscopic CTR, 21 days for mini-open CTR, and 31 days for open CTR.^34^ Nearly 3 in 10 Americans skip medical treatment due to cost, and 37% cannot cover an unexpected $400 expense.^35^ Thus, missing even 1 week of work can create substantial financial hardship. Returning patients to normal function and productivity within days rather than weeks through office-based CTR-US may avoid prolonged disability and lost wages, aligning with US Department of Labor policies promoting rapid return to work after illness or injury.^36^

Another advantage of office-based CTR-US is the ability to perform simultaneous bilateral procedures during a single visit, rather than two-staged procedures in an ASC or hospital. Patients may benefit from reduced costs, time commitment, and recovery when treating both hands at the same clinic visit. With office-based simultaneous bilateral CTR-US, patients can promptly address symptoms in both hands and resume daily activities and work without staging delays or doubling facility costs. For appropriate candidates, office-based simultaneous bilateral CTR-US may optimize clinical and economic value compared with staged facility-based procedures.

Office-based surgery may raise concerns about sterility, anesthesia, and emergency backup compared with traditional operating rooms. However, accumulating evidence with various CTR techniques,^9^, ^10^, ^11^, ^12^ including results from this multicenter study, challenge these concerns. In the current study, no intraoperative complications were noted, and a single complication was identified during follow-up among 226 treated hands. Despite nearly one in four patients having anxiety before surgery, all were successfully treated with CTR-US using WALANT with minimal intraoperative pain. The complication rate in this trial (0.4%) was comparable with other studies of CTR-US^16^^,^^21^ and both endoscopic and mini-open CTR^37^ performed in mixed settings. Thus, this prospective multicenter study establishes office-based CTR-US as a safe procedure, further contributing to the growing evidence base supporting the safety of office-based CTR.^9^^,^^12^

Performing CTR-US in an office versus a facility setting may reduce costs for surgeons and payers. For surgeons, office-based procedures may reduce facility fees, surgical suite expenses, staffing, anesthesia, and other overhead costs of facility-based procedures. Office procedures may also allow for more efficient use of surgeon and staff time by scheduling minor procedures like CTR-US between clinic visits rather than blocking large time slots for facility-based surgery. For payers, the total cost is substantially lower with office-based versus facility-based procedures. One study reported a 46% reduction in the 90-day postoperative costs with minor hand procedures (predominantly CTR) performed in an office versus outpatient hospital setting and a 34% reduction compared with ASCs.^12^ With 600,000 CTRs performed annually nationwide,^3^ shifting procedures from facilities to offices could translate into significant cost savings of approximately $600 million annually based on these projections.

There are some limitations to consider when interpreting these study results. First, this study reports follow-up data through 6 months. Although most complications and clinical improvements occur during this period,^38^ longer follow-up will determine the durability of symptom relief and potential for late complications. Subjects in the current trial will be followed for 2 years, after which final results will be reported. Second, this was a prospective case series with no control group. Thus, indirect outcome and cost comparisons to other CTR techniques and settings should be interpreted cautiously. Third, participants elected to receive office-based CTR-US after consultation with the treating surgeon, which may introduce potential bias in patient-reported outcome measures. Finally, CTR-US using WALANT may not be appropriate for certain patients. Patients who are uncomfortable with being awake during surgery or who are at higher risk for local anesthesia reactions due to vasculopathy or hypersensitivity may be poor candidates for WALANT.

In conclusion, this prospective multicenter study demonstrated that office-based CTR-US, performed either unilaterally or as simultaneous bilateral procedures, is well tolerated with a low complication rate and associated with rapid recovery, sustained improvement in symptoms and function, and high procedure satisfaction. The 6-month results of the ROBUST trial support office-based CTR-US as a safe and effective alternative to facility-based procedures.

## Conflict of Interest

Drs Paterson and Miller report consultancy with Sonex Health, Inc. Drs Pistorio, Marwin, Alexander, and Nelson report no conflicts of interest.
