# Supplementary material for: Office-Based Carpal Tunnel Release With Ultrasound Guidance: 6-month Outcomes From the Multicenter ROBUST Trial
Source: J Hand Surg Glob Online. 2024 Feb 19;6(3):268–74. doi: 10.1016/j.jhsg.2023.12.005 (PMC11133848; doi:10.1016/j.jhsg.2023.12.005)
Supplement: Supplementary Tables [file mmc1.docx]

**SUPPLEMENT**

**Supplement Table 1. List of oversight committees and investigators in the ROBUST trial.**

| **Name** | **Institution** | **Location** |
| --- | --- | --- |
| **Data safety monitoring board** |  |  |
| Kevin C. Chung, MD, MS | University of Michigan Health | Ann Arbor, MI |
| Julie E. Adams, MD | University of Tennessee College of Medicine | Chattanooga, TN |
| Warren C. Hammert, DDS, MD | Duke University | Durham, NC |
| **Independent medical reviewer** |  |  |
| Kevin C. Chung, MD, MS | University of Michigan Health | Ann Arbor, MI |
| **Principal investigator** |  |  |
| Ashley L. Pistorio, MD, MD | University of Nevada, Las Vegas | Las Vegas, NV |
| **Trial investigators** |  |  |
| Randall D. Alexander, MD | Georgia Hand, Shoulder & Elbow | Atlanta, GA |
| Victor M. Marwin, MD, MBA | Bluegrass Orthopaedics | Lexington, KY |
| Johnny T. Nelson, MD | The Bone and Joint Surgery Clinic | Raleigh, NC |
| Paul D. Paterson, MD | Vero Orthopaedics | Vero Beach, FL |
| Richard Jordan Post, MD | New Braunfels Orthopaedic & Sports Medicine | New Braunfels, TX |
| James F. Watt, DO | Orthopedic Associates | Tampa, FL |

**Supplement Table 2. Subject eligibility criteria.**

| Inclusion Criteria |
| --- |
| 1. ≥18 years of age |
| 1. Clinical diagnosis of unilateral or bilateral idiopathic CTS |
| 1. CTS-6 score ≥12 in target hand* |
| 1. Median nerve cross-sectional area ≥10 mm2 in the proximal carpal tunnel region of the target hand measured by diagnostic ultrasound* |
| 1. Prior failure of one or more nonsurgical treatment options (e.g., physical activity modification, bracing, splinting, corticosteroid injection)* |
| 1. Subject agrees to complete follow-up questionnaires over a 24-month period |
| 1. Subject has a valid smart phone number and/or email address to receive and answer follow-up questionnaires |
| Exclusion Criteria |
| 1. Prior surgery on the target wrist or hand with the exception of (a) trigger finger release or similar minor finger procedure (e.g., digital ganglion cyst removal, foreign body removal) that has clinically recovered, or release for DeQuervain's syndrome (1^st^ dorsal compartment) that has clinically recovered* |
| 1. History of prior surgical CTR in the target hand* |
| 1. History of infection in the target hand* |
| 1. History of prior surgery in the non-target hand, including CTR, within 3 months of enrollment or with persistent symptoms that interfere with normal daily activities or work at the time of consent |
| 1. Planned surgical or interventional procedure on the contralateral hand within 3 months of the target hand procedure date* |
| 1. Corticosteroid injection in the target hand within 6 weeks of study procedure date* |
| 1. Presence of additional process in the target hand requiring additional intervention beyond carpal tunnel release (e.g. neurolysis, mass removal, tenosynovectomy)* |
| 1. Clinically significant** degenerative arthritis of the upper limb (shoulder to hand) on the target side* |
| 1. Clinically significant** inflammatory disease (including tenosynovitis) of the upper limb (shoulder to hand) on the target side |
| 1. Clinically significant** trauma or deformity of the upper limb (shoulder to hand) on the target side* |
| 1. Clinically significant** vascular disease (including Raynaud's phenomenon) of the upper limb (shoulder to hand) on the target side* |
| 1. Clinically significant** neurological disorder (including complex regional pain syndrome) of the upper limb (shoulder to hand) on the target side* |
| 1. Systemic inflammatory disease (e.g., rheumatoid arthritis, lupus) |
| 1. Amyloidosis |
| 1. Chronic renal insufficiency requiring dialysis |
| 1. Diabetes not controlled by a stable dose of medication |
| 1. Uncontrolled thyroid disease |
| 1. Pregnant or planning pregnancy in the next 24 months |
| 1. Workers' compensation subjects |
| 1. Inability to provide a legally acceptable Informed Consent Form and/or comply with all follow-up requirements |
| 1. Subject has other medical, social, or psychological conditions that, in the opinion of the investigator, preclude them from receiving the pre-treatment, required treatment, and post-treatment procedures and evaluations |

*Criterion must be applied to the target hand for unilateral CTR-US procedures, or to both hands for simultaneous bilateral CTR-US procedures.

**Clinically significant is defined as likely to interfere with the performance of the procedure in a safe and/or effective manner.

**Supplement Table 3. Study assessments at each follow-up interval.**

| **Assessment** | **Baseline** | **Procedure** | **Post-Op** | **Daily**  **1-14** | **Months** | | | | |
| --- | --- | --- | --- | --- | --- | --- | --- | --- | --- |
|  |  |  |  |  | **1** | **3** | **6** | **12** | **24** |
| **Site Assessments** |  |  |  |  |  |  |  |  |  |
| Demographics | **■** |  |  |  |  |  |  |  |  |
| Ultrasound median nerve cross-sectional measurement | **■ *** |  |  |  |  |  |  |  |  |
| CTS-6 | **■ **** |  |  |  |  |  |  |  |  |
| Procedure |  | **■** |  |  |  |  |  |  |  |
| Adverse events |  | **■** | **■** | **■** | **■** | **■** | **■** | **■** | **■** |
| **Subject-reported Outcomes** |  |  |  |  |  |  |  |  |  |
| Demographics | **■** |  |  |  |  |  |  |  |  |
| Medical history | **■** |  |  |  |  |  |  |  |  |
| BCTQ-SSS | **■** |  |  | **■ ***** | **■** | **■** | **■** | **■** | **■** |
| BCTQ-FSS | **■** |  |  | **■ ***** | **■** | **■** | **■** | **■** | **■** |
| MHQ | **■** |  |  | **■ ***** | **■** | **■** | **■** | **■** | **■** |
| Numeric Pain Scale | **■** | **■** | **■** | **■** | **■** | **■** | **■** | **■** | **■** |
| EQ-5D-5L | **■** |  |  | **■ ***** | **■** | **■** | **■** | **■** | **■** |
| Global satisfaction |  |  |  | **■ ***** | **■** | **■** | **■** | **■** | **■** |
| Procedure |  | **■** |  |  |  |  |  |  |  |
| Return to activities |  |  |  | **■** | **■** | **■** | **■** | **■** | **■** |
| Return to work † |  |  |  | **■** | **■** | **■** | **■** | **■** | **■** |
| Pain medication | **■** | **■** | **■** | **■** | **■** | **■** | **■** | **■** | **■** |

*Performed on all treated hands.

**Measured on both hands.

***Collected at 14-day evaluation only.

†Collected on employed subjects only.

BCTQ-FSS, Boston Carpal Tunnel Questionnaire Functional Status Scale; BCTQ-SSS, Boston Carpal Tunnel Questionnaire Symptom Severity Scale; EQ-5D-5L, EuroQoL-5 Dimension 5-Level; MHQ, Michigan Hand Questionnaire.
